# Integrating remote blood pressure monitoring into NHS primary care: a human factors perspective

**DOI:** 10.3389/fdgth.2026.1697787

**Published:** 2026-03-05

**Authors:** Massimo Micocci, Omar Butt, ShanShan Zhou, Austen El-Osta, Peter Buckle, George B. Hanna

**Affiliations:** 1 NIHR HRC IVD, Department of Surgery and Cancer, Imperial College London, London, United Kingdom; 2 Self-Care Academic Research Unit (SCARU), School of Public Health, Imperial College London, London, United Kingdom

**Keywords:** digital health, human factors, hypertension management, implementation barriers, remote monitoring, workflow efficiency

## Abstract

**Background:**

Hypertension remains a major health burden in the UK, contributing significantly to cardiovascular disease and health inequalities. Although digital health technologies offer opportunities to enhance hypertension management, current NHS pathways face challenges, including inefficiencies in patient monitoring, limited patient engagement, and resource constraints. This study aimed to evaluate integration challenges of remote digital monitoring tools for blood pressure into NHS hypertension care pathways.

**Methods:**

An exploratory study combining semi-structured interviews with 14 primary care NHS stakeholders recruited from across England, and a field study at two GP practices. Participants were selected to have either experience or not with digital platforms for remote monitoring of chronic conditions in primary care. Clinical pathway mapping and gap analysis were used to identify inefficiencies in hypertension management and explore digital platforms’ potential integration.

**Results:**

Eight major gaps were identified, including inconsistent patient engagement, lack of automated identification of at-risk and non-compliant patients, limited access to home monitors, and health inequalities related to digital literacy. Integration of a digital platform addressed several of these gaps by promoting self-monitoring behaviours, improving resource allocation through risk stratification, and enhancing decision-making with continuous patient data. However, barriers such as interoperability issues, workload concerns, literacy disparities, and unclear role responsibilities were noted.

**Conclusion:**

Successful implementation requires addressing systemic challenges through targeted training, robust interoperability standards, clearer task allocation, and equity-focused interventions to bridge the digital divide. A human-centred, system-wide strategy is essential to ensure sustainable adoption and maximise the impact of digital innovations in primary care.

## 1 Introduction

The National Health Service (NHS) in England is facing a mounting challenge due to the rising burden of long-term conditions (LTCs), which are projected to significantly increase by 2040 (1). Cardiovascular disease (CVD) remains a leading contributor to morbidity, disability, and mortality in the UK, affecting approximately seven million people and imposing a considerable economic burden on the health system (2, 3). To mitigate these pressures, the expansion of independent prescribing rights to pharmacists since 2006—and forthcoming mandates for universal prescribing capability among newly qualified pharmacists by 2026—reflect a strategic shift toward multidisciplinary models of care within community settings (4). This transformation necessitates adaptable integration pathways and user training to accommodate evolving digital infrastructures. Within this context, digital health has gained prominence through national strategies such as the NHS “Fit for Future—10 Year Health Plan for England” released in July 2025 (5), emphasising a fundamental transformation from analogue to digital systems, enabling more integrated, data-driven, and patient-centred models of care. The rapid emergence of digital therapeutics and artificial intelligence (AI) further expands this potential by enabling evidence-based digital interventions and predictive analytics to support more proactive, personalised, and efficient models of care (6, 7). The COVID-19 pandemic triggered a rapid, necessity-driven increase in the adoption and acceptance of digital health technologies and remote monitoring, facilitated by temporary regulatory changes; however, sustaining long-term integration remains challenging due to persistent digital divides, infrastructure limitations, declining post-pandemic momentum, and socio-demographic differences in access, trust, and perceptions of privacy and data security (8, 9) and socio-demographic differences in access, trust, and perceptions of privacy and data security (10).

The NHS is currently trialling several digital platforms and strategies, e.g., the BP@home program (11) to support remote monitoring of hypertension across primary care. At the time of this investigation, the Viso platform from OMRON Healthcare was being increasingly adopted by GP practices for remote blood pressure monitoring. This system was often used in coordination with the Accurx digital communication platform, which integrates with the Electronic Health Record (EHR) to enable secure messaging with patients about appointments and care-related communications.

Digital health tools have demonstrated significant potential to increase patient activation—defined as an individual's knowledge, skills, and confidence in managing their own health. Evidence shows that users of digital health platforms achieve higher Patient Activation Measure (PAM) scores, which are strongly associated with better treatment adherence and improved health outcomes (12, 13). Recent studies demonstrate the growing role of digital and AI-enabled approaches in hypertension management; a WeChat-based multimodal digital intervention significantly improved blood pressure control and quality of life compared with usual care (14), while a large language model (LLM)–based clinical agent showed potential to support clinician decision-making and workflow efficiency in managing suspected hypertension (15); additionally, long-term evidence from self-monitoring and self-titration interventions indicates that patient-centred remote management strategies can sustain blood pressure reductions without increasing healthcare utilization (16).

However, healthcare professionals report challenges including a lack of condition-specificity, poor integration with existing NHS IT systems, insufficient digital literacy among users and staff, time constraints, limited evidence bases, and data security concerns (17). Digital literacy remains uneven across demographics, with older adults and certain populations lagging (18). Poor user interface design, for instance, results in tools that are difficult for healthcare providers and patients to navigate, leading to inefficiencies and errors (19). Furthermore, many digital health solutions disrupt existing clinical workflows rather than integrating seamlessly into them, creating additional burdens for already overstretched healthcare professionals (20).

Furthermore, resource allocation has paradoxically favoured hospitals, with their share of NHS spending rising from 47% in 2006 to 58% in 2021 (6). The transition is impeded by the underutilisation of existing digital assets, the absence of essential workflow technologies within community services, and the lack of a clearly defined pathway for the integration of these tools into routine practice.

This exploratory study aims to provide a comparative qualitative evaluation (adopters vs. non-adopters of the perceived barriers and implementation aspects for remote monitoring tools for hypertension within NHS primary care. The study seeks to understand the Human Factors principles that facilitate or hinder a smooth transition to the digitalisation of long-term condition monitoring in the NHS, while identifying key roadblocks to implementation and generating evidence to inform strategies for successful adoption. Specifically, we sought to:

Map current hypertension management pathways to identify workflow inefficiencies and unmet needs.
Assess how digital remote monitoring can address these gaps, including its role in risk stratification, self-monitoring, and clinical decision-making as perceived by adopters vs. non-adopters of existing digital platforms.
Identify human factors, usability issues, and systemic barriers—such as interoperability challenges, workload implications, and digital literacy disparities—that influence successful adoption.

## 2 Methods

### 2.1 Study design

This study employed qualitative research methods to evaluate the current standard of care for hypertension management and explore potential changes associated with integrating remote monitoring tools into primary care. Two main activities were undertaken:

**Stakeholder interviews**—We conducted 40–45 min semi-structured remote interviews (via Zoom or Microsoft Teams) with primary care stakeholders to map current workflows for monitoring hypertension patients. Interview questions addressed key components of the work system—tasks, environments, regulatory factors, and personnel (21, 22) as well as existing bottlenecks, mitigation strategies, and anticipated changes following implementation. Stakeholders’ feedback was shaped by their personal experience and familiarity (or lack thereof) with digital monitoring tools for hypertension. The adopters were all users of Viso, a digital health solution designed by OMRON Healthcare for remote blood pressure monitoring and hypertension management. The platform comprises a healthcare professional dashboard and a patient-facing mobile interface. GP practices identify eligible patients based on their clinical history and chronic conditions, then onboard them onto the digital system. Once enrolled, patients independently measure their blood pressure and upload readings via the mobile interface. Healthcare professionals—including GPs, specialist nurses, and clinical pharmacists with independent prescribing authority—access these readings through a dedicated dashboard that integrates directly with the Electronic Health Record (EHR). Instances of patient non-compliance automatically trigger alerts on the clinician dashboard, enabling timely reminders or interventions. Following each submission cycle, prescribers review the data and, where necessary, adjust medication regimens through titration to optimise blood pressure control.
**Field studies with current users**—A think-aloud protocol systematically asks participants to verbalise their thoughts, decisions, and reasoning in real time while performing representative tasks, enabling researchers to capture cognitive processes, usability issues, and workflow challenges without prompting or interpretation bias. Participants performed core tasks including: checking notifications for patients requiring review, creating medication plans using EHR data, reviewing recent blood pressure measurements, consulting platform recommendations, ordering further tests, cross-checking information on the EHR, and monitoring medication compliance. Cognitive demand was operationalised by systematically recording task interruptions, unnecessary switching between systems, instances of missing or incomplete information that required additional actions, and moments of misinterpretation or uncertainty when interacting with the platform, allowing us to identify points of increased mental workload during task execution. Each session, conducted *in situ* by two researchers (MM and OB), lasted approximately one hour and was followed by a short interview to explore cognitive demands, system interoperability, equipment use, user workload, data sharing with GP practices, and IT-related issues.

The semi-structured interview guide was divided into two main sections. In the first part, participants were asked about the current clinical workflow for monitoring patients with hypertension -pre digital platform implementation-, including patient entry points, diagnostic processes, remote monitoring, medication adjustments, care review timelines, and perceived gaps in the current pathway. In the second part, the questions diverged according to participants' experience with remote monitoring tools:

**For adopters** of the Viso platform, questions explored real-world experience, including how the technology had been used, its integration into existing workflows, perceived impact on treatment decisions, advantages and disadvantages, barriers and facilitators to adoption, and overall confidence in the platform's contribution to patient care.
**For non-adopters**, we provided a short presentation outlining the key functionalities of remote monitoring for hypertension, including a description of Viso's core features. Participants were then asked to reflect on how such a system could be integrated into their clinical workflow, anticipated benefits, perceived barriers, feasibility, trustworthiness, and factors influencing potential adoption (e.g., interoperability, staff readiness, patient engagement).

### 2.2 Participants

Participants were recruited using purposive sampling to ensure representation of diverse professional roles involved in hypertension management in NHS primary care. We leveraged our professional network within primary care to access contact details of potential participants across England. The final sample comprised 14 NHS stakeholders: General Practitioners (*n* = 6, codes GP01–GP06), Clinical Pharmacists (*n* = 4, codes CP01–CP04), a Nurse Practitioner (NP01), a Digital Care Coordinator (DCC01), an IT Specialist (IT01), and a Healthy Lifestyle Action Programme Facilitator (HP01). Participants were classified into two groups based on their prior experience with remote blood pressure monitoring:

**Adopters** (*n* = 5): stakeholders who were already using Viso in their practice.
**Non-adopters** (*n* = 9): stakeholders who had never used remote blood pressure monitoring tools.

No other background knowledge, experience, or demographic characteristics were considered in the eligibility criteria beyond professional role and technology adoption status.

Among Viso adopters, we have engaged with two users to conduct the field study. The study was conducted with one Clinical Pharmacist (CP02—Practice 1) and one Nurse Practitioner (NP01—Practice 2). Both were independent prescribers. Participation in the observation was limited to these two individuals, as they were the only stakeholders who agreed to take part. The recruitment process for both activities included distributing study invitations via email, which contained a Participant Information Sheet, Consent Form, and references to ethical approval. Participant recruitment focused on capturing variation between adopters and non-adopters, as well as diversity across relevant stakeholder groups. Given the exploratory nature of this pilot study, the aim was not statistical generalisation but to ensure sufficient breadth of perspectives to identify recurring themes. Participants were pseudonymized, and each was assigned a unique, progressively numbered code to facilitate data analysis while maintaining confidentiality. This study, involving human participants, was reviewed and approved by Imperial College Research Ethics Committee and received a favourable opinion (ICREC 6711349). Written informed consent to participate in this study was provided by the participants.

### 2.3 Data analysis

Data analysis is examined by multiple researchers to enhance interpretive rigour and reduce individual bias (MM, OB). The process included summarising and thoroughly reading the data, followed by open-coding of transcripts. Codes were deliberated upon to establish consensus, and themes were identified through constant comparison until saturation was reached. Interviews were audio-recorded and transcribed verbatim for analysis using NVivo software (v.12, QSR). The analysis involved a thematic approach, with data organised using the Framework Analysis Method (FAM) to support thematic analysis (23). Clinical Pathway Mapping (CPM) was utilised to understand the patient journey and its impact on NHS resources and patient outcomes (24–27). A gap analysis was conducted to identify discrepancies between the current state of care processes and ideal or best practice standards. This analysis helped in pinpointing areas where improvements are needed, such as inefficiencies in care delivery, gaps in patient safety, or deviations from established guidelines (28).

## 3 Results

### 3.1 Clinical pathway and gap analysis

**Figure 1:**
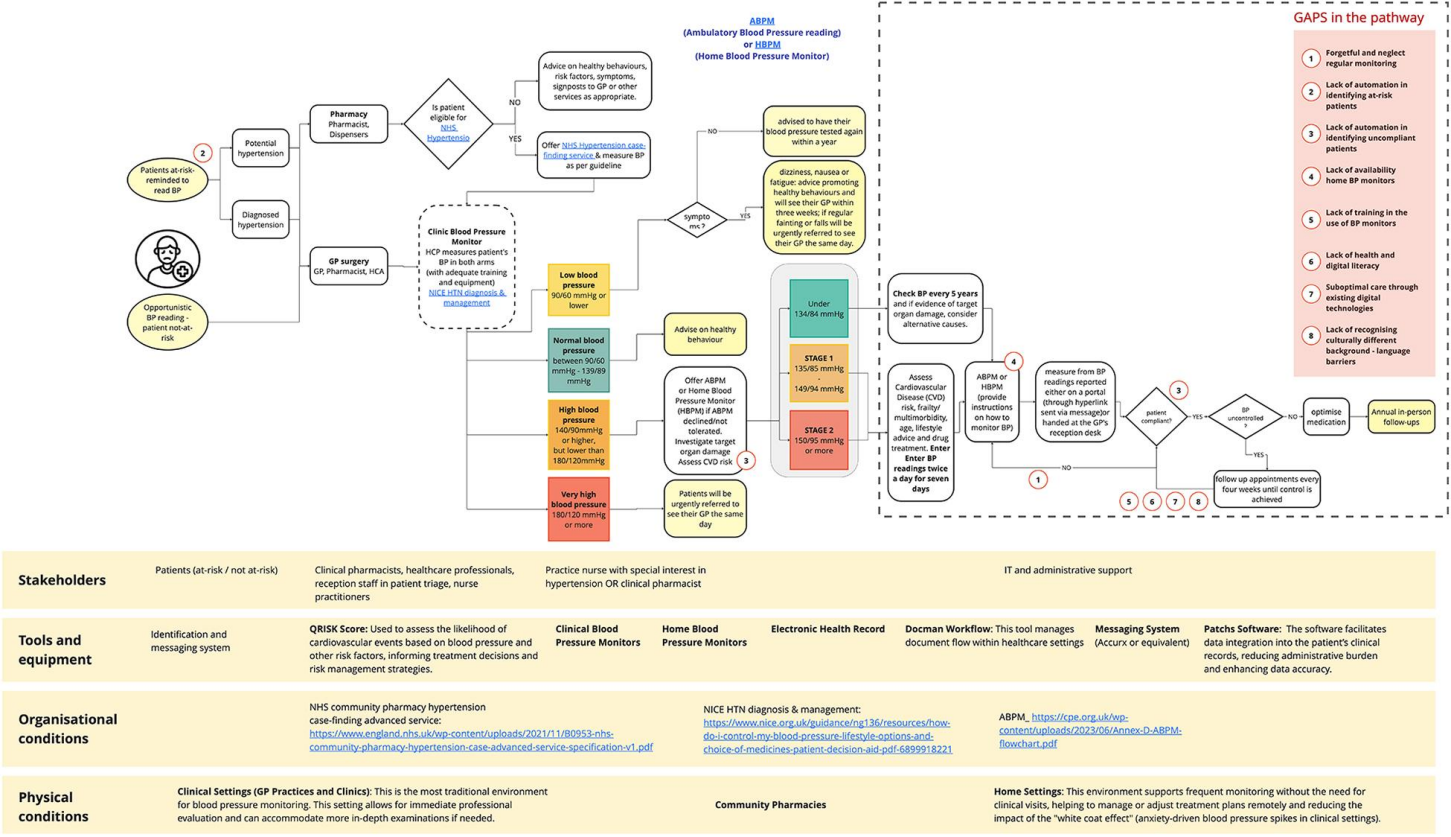
Conventional NHS hypertension pathway with the eight identified workflow bottlenecks.

The current clinical workflow revealed several gaps and bottlenecks that hinder effective patient care (highlighted by dashed areas in Figure 1). These challenges are concentrated primarily in the latter part of the pathway, following patient onboarding and during the monitoring phase. The initial stage of hypertension management in primary care follows well-established processes, beginning with multiple entry points such as GP appointments, pharmacy referrals, and home monitoring programs, supported by reception triage and clinical pharmacists with independent prescribing authority. Patient classification adheres to NHS guidance, with individuals presenting blood pressure readings below 140/90 mmHg monitored every five years, and those with borderline or high-risk profiles—such as comorbid diabetes—undergoing more frequent Cardiovascular Risk Score (QRISK) assessments and annual reviews. In contrast, the later phase of the pathway is characterised by unpredictability in patient monitoring and non-adherence to recommended follow-up, representing the main source of inefficiency identified by participants. This clinical pathway typically requires 6–24 months for stabilisation in newly diagnosed patients, with intervention frequency calibrated to blood pressure control status. Bottlenecks of the current clinical pathway are listed as follows:

**Forgetful and neglect regular monitoring**: Many patients fail to maintain consistent monitoring without direct reminders. Multiple healthcare professionals (GP01, CP03) noted that appointment availability and patient prioritisation affect compliance.
**Lack of automation in identifying at-risk patients**: The system lacks effective methods to proactively identify individuals with undiscovered hypertension, though community pharmacy case-finding services show promise.
**Lack of automation in identifying uncompliant patients**: No efficient system flags medication non-adherence despite technical capabilities existing (GP04, NP01). High-risk patients often engage least with healthcare.
**Lack of availability of home BP monitors**: Cost barriers and NHS resource limitations create inequitable access to monitoring devices (CP03, GP03, GP05).
**Lack of training in BP monitor use**: Patients receive inadequate guidance on proper monitor usage despite its critical importance for accurate readings (GP04, GP05).
**Lack of health and digital literacy**: Socioeconomic factors influence digital engagement, with older patients and those from disadvantaged backgrounds struggling with technology adoption (IT01, GP03).
**Suboptimal care through existing digital technologies**: Current NHS app capabilities are limited for remote patient monitoring (GP03).
**Lack of recognising culturally different backgrounds**: Language barriers and cultural insensitivity exclude certain patient populations from effective engagement (GP04).

**Figure 2:**
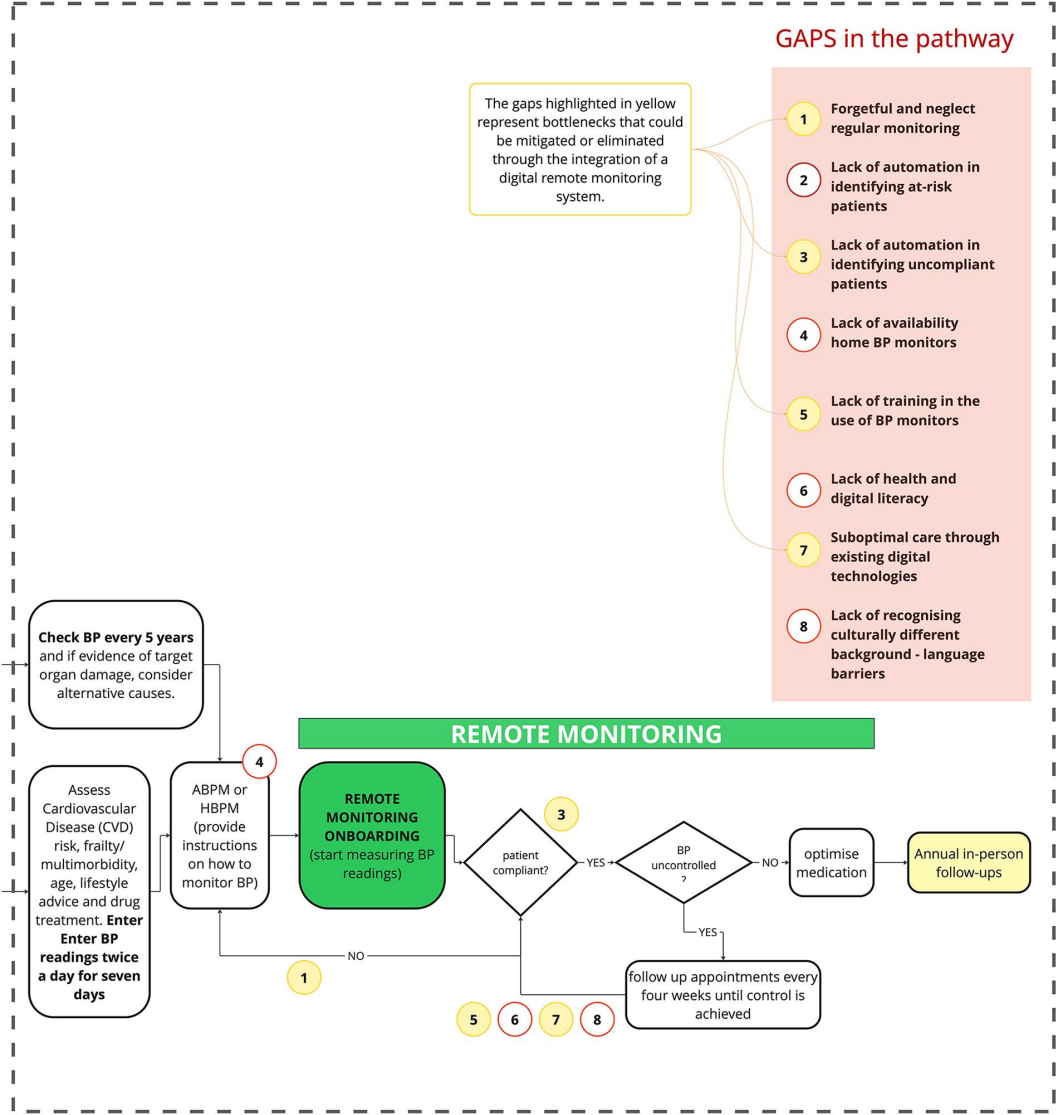
Hypertension pathway with digital remote monitoring integration; yellow areas indicate mitigated bottlenecks.

The introduction of a remote monitoring tool has brought notable changes to the conventional hypertension management pathway among the adopter group (Figure 2). Discussions with adopters indicated that the use of such a tool could substantially reduce the number of workflow bottlenecks—from eight to four—by streamlining monitoring and follow-up processes. The bottlenecks that could be mitigated or eliminated through the integration of a digital remote monitoring system are highlighted in yellow in Figure 2. Remaining challenges centred on the limited availability of home blood pressure monitors, disparities in digital literacy among older adults, and the need for enhanced patient education during implementation. These perspectives were largely echoed by non-adopters, despite their lack of direct experience with remote monitoring platforms. These conclusions emerge from the thematic analysis, with the key themes synthesised into three main use cases outlined in Table 1. In summary, participants advocated for empowering patients by enhancing their understanding of blood pressure readings and providing personalised guidance for self-management. They emphasised the importance of building health literacy through educational content, promoting proactive health behaviours, fostering patient ownership of condition management, and establishing a positive feedback loop that reinforces engagement. Moreover, participants highlighted the critical role of automating patient identification and prioritisation processes to reduce manual administrative burdens, improve resource allocation through data-driven patient management, and streamline clinical workflows by enabling systematic monitoring, efficient tracking, and follow-up. This approach supports the development of sustainable operational processes within primary care settings. Finally, the continuous monitoring enabled by the digital tool was recognised as a valuable asset for generating reliable data to support clinical decision-making, reducing risk through the timely detection of concerning readings, enabling evidence-based treatment adjustments, and allowing care plans to be dynamically adapted in response to real-time patient data. This capability enhances the quality, safety, and consistency of care, enabling timely interventions before complications arise. Each use case has been associated with three distinct domains of intervention: the patient (personal) domain, the administrative domain, and the clinical domain.

**Table 1:** Summary of benefits in the use of the digital platform for remote monitoring of hypertension and the three use cases identified.

Domain	Personal/Patient	Administrative	Clinical
Use cases	Facilitating Enhanced Health Literacy and Proactivity	Streamlining Patient Monitoring and Optimising Staff Allocation	Enhancing Clinical Decision-Making and Patient Care
Description	Empowering patients with knowledge about their blood pressure readings Providing personalised guidance on managing their condition Building health literacy through educational content Encouraging self-management and proactive health behaviours Supporting patients to take ownership of their condition Creating a feedback loop that reinforces positive health actions	Automating patient identification and prioritisation processes Reducing manual administrative workload for healthcare staff Improving resource allocation through data-driven patient management Streamlining workflow through systematic patient monitoring Enabling efficient patient tracking and follow-up Creating sustainable operational processes	Providing reliable, continuous data for informed treatment decisions Reducing clinical risks through timely identification of concerning readings Enabling evidence-based modifications to treatment plans Supporting dynamic adaptation of care based on patient data Improving the quality and consistency of clinical care Facilitating timely interventions before complications develop
Expected outcome	Enhanced engagement with the platform and improved hypertension management, reducing the need for extensive secondary care and lowering healthcare costs while improving patient outcomes	Improved access to care and overall health service delivery, with more efficient allocation of healthcare resources	Streamlined operations and improved the effectiveness of healthcare delivery through better-informed clinical decisions

### 3.2 Challenges and barriers to implementation

The integration of digital technologies for remote monitoring into existing hypertension management pathways presents several key challenges alongside potential implementation strategies. These considerations span both individual user experience and system-level factors, with notable differences between early and late adopters. Overall, adopters reported that the platform improved patient monitoring and clinical decision-making but noted challenges such as “toolbar fatigue,” duplicative documentation, and the need for continual patient reminders. They emphasised that larger practices with more resources were better positioned for successful implementation. In contrast, non-adopters voiced scepticism about the effectiveness of digital platforms for patients with low engagement or limited digital literacy, raised concerns over increased workload and potential safety risks from manual data entry, and emphasised the need for seamless integration with existing EHR systems. A summary of the challenges discussed by the two groups of participants is presented in Table 2.

**Table 2:** Summary of the challenges identified and discussed by both Adopters and Non-adopters.

Challenges	Adopters	Non-adopters
User Acceptance Challenges	Successful implementation hinges on demonstrating local evidence of seamless deployment to build trust (GP02). Patient engagement varies significantly, with some requiring frequent reminders for self-monitoring (HP01, DCC01). The platform faces adoption barriers, including account creation difficulties, particularly for older patients (GP05), interface complexity, and competition from established solutions	Scepticism was expressed regarding Viso's effectiveness for individuals with inconsistent health management habits (CP01), particularly those frequently transitioning between care settings. Complex data entry requirements may deter adoption, especially in practices managing numerous hypertension patients (CP03)
Health Inequalities Concerns	They note the platform risks introducing care biases, with more engaged patients potentially receiving disproportionate attention while less digitally inclined individuals become unintentionally neglected (GP02). The platform doesn't inherently increase monitoring participation as it relies on patient initiative	They emphasise concerns about digital literacy requirements excluding elderly patients (GP05) and suggest positioning Viso within annual review processes led by nursing teams or clinical pharmacists (GP04)
Workload Management Issues	They reported “toolbar fatigue” from managing multiple digital platforms simultaneously (GP05) and duplicative documentation burdens when updating patients’ data on both systems, the digital platform and electronic health records (HP01). Following up with non-participating patients proves time-consuming (DCC01)	They expressed concerns about increased workload from managing patient-reported anomalous readings (CP03) and questions regarding data management responsibilities, checking frequency, and clinical liability
Responsibility and Task Allocation	They acknowledged that larger practices have more resources to effectively deploy the platform (GP02)	They observed that implementation success varies significantly by Primary Care Network funding and pharmacy support levels (CP01). Many general practitioners face overwhelming pressure in meeting core patient needs, making new technology adoption risky despite potential benefits (GP04). While a remote monitoring tool could centralise clinical pharmacist workflows, it risks compartmentalising hypertension management rather than maintaining it as a team-wide responsibility
System Integration and Interoperability	They reported unclear data provenance in patient records (CP02) and perceived inconsistencies between the platform and electronic health records, leading to patient confusion (NP01)	They emphasise safety risks associated with manual information entry (GP04) and the need for seamless integration with existing electronic health record systems, automated identification of patients with suboptimal readings, and single login functionality

### 3.3 Field study

The two GP practices involved in the field demonstrated alignment in several areas. Both experienced synchronisation challenges between the new platform and legacy IT systems, with data transfer delays creating workflow inefficiencies. They shared concerns about incomplete patient data transfer from EHRs to the platform, particularly regarding ethnicity information, which could impact treatment decisions. Both practices emphasised the importance of human judgment in reviewing system-generated recommendations before approval. However, notable differences emerged in their operational approaches. Practice 1 highlighted specific difficulties with finding recent recommendations in the platform interface, requiring additional steps to view complete details. Practice 2 maintained a tracking system to monitor patient engagement metrics, including time spent with each patient. Additionally, Practice 2 expressed more concerns about how IP (Independent Prescriber) comments appeared in the EHR, noting confusion about who was reviewing patients when modifications came from the platform. We have summarised the key interaction tasks and feedback from both practices to explore usability issues. These are further classified based on the requirements of Effectiveness, Efficiency and User Satisfaction, as per ISO 9241-11:2018:

**Effectiveness:** Both practices reported data synchronisation issues between the platform and current EHR systems, occasionally resulting in incomplete or erroneous patient information. The platform also showed limitations in efficiently identifying and registering eligible patients, particularly those at higher risk or with specific health conditions.
**Efficiency**: Both practices identified limitations in the notification process, particularly the lack of alerts when test results were returned, which slowed the care process. Manual data updates or checks were frequently required, as the platform didn't always quickly reflect changes in the EHR.
**User satisfaction**: While the platform interface was considered generally user-friendly, the necessity to use multiple screens or platforms to verify and update patient information complicated the user experience. The platform also lacked significant features for direct monitoring of medication compliance, with healthcare practitioners having to infer non-compliance through indirect methods like medication reorder patterns.

## 4 Discussion

This study highlights the challenges and opportunities associated with integrating digital health technologies into hypertension management pathways. The findings emphasise systemic issues such as the digital divide, inefficiencies in workflows, interoperability challenges, and the need for training and role clarity. Additionally, gaps in the current hypertension care pathway—such as the lack of risk stratification, systematic follow-up, and resource allocation—underscore the need for innovative solutions. The need for improved care coordination and patient self-management support is crucial to address these challenges. Below, these aspects are discussed comprehensively.

### 4.1 Digital divide and socioeconomic barriers

The digital divide continues to hinder equitable access to healthcare technologies. Socioeconomic and cultural factors significantly influence patients' engagement with digital platforms, particularly among older adults and those from lower-income groups who often face barriers such as limited digital literacy or access to technology (18, 19). Research highlights that disparities in internet access and digital health literacy exacerbate inequities in healthcare outcomes, disproportionately affecting vulnerable populations and individuals with lower socioeconomic status (29, 30). Bridging this gap requires targeted interventions, including simplified interfaces, culturally sensitive education materials, and tailored support programs (13). Studies emphasise that addressing social determinants of health and promoting equity through infrastructure development and policy changes are critical to reducing the digital divide (31, 32).

### 4.2 Efficiency concerns in clinical pathways

Digital platforms can effectively address inefficiencies in healthcare by using data-driven algorithms to identify high-risk patients early, enabling timely interventions that improve outcomes and reduce costs. These platforms also support optimised resource allocation, enhancing operational efficiency and care delivery across healthcare systems (33–35). However, the lack of patient stratification and prioritisation leads to inefficiencies in patient care (17).

### 4.3 Interoperability challenges

Healthcare interoperability remains a major challenge due to fragmented IT systems, incompatible data standards, and diverse workflows across organisations, which often leads to disjointed care and undermines the potential of digital health technologies (36, 37). Achieving interoperability between systems requires addressing multiple interconnected dimensions (38, 39): syntactic interoperability ensures that health data are consistently structured and formatted for exchange [using international standards development organizations (SDOs) such as Health Level Seven International—HL7- or Integrating the Healthcare Enterprise—IHE], while semantic interoperability guarantees that the meaning of the data is universally understood across systems through standardized terminologies like SNOMED CT (39); implementing these standards, alongside organizational alignment on shared policies, is essential for seamless data sharing, patient safety, and the effective integration of innovations such as AI, Internet of Medical Things (IoMT), and telemedicine platforms with EHR (37, 40).

### 4.4 Role definition and resource allocation

A clear role definition is essential for the successful implementation of digital health technologies within complex care pathways. For instance, reception staff play a pivotal role in triaging patients but often face unclear responsibilities that lead to inefficiencies (17). Similarly, inconsistent availability of blood pressure monitors highlights the need for better resource allocation and training to support both patients and healthcare providers.

### 4.5 Importance of training and familiarity

Healthcare professionals often lack sufficient training in using remote monitoring tools effectively, undermining their adoption and resulting in workflow inefficiencies (41). Comprehensive education programs tailored to different user groups—patients, reception staff, and clinicians—are essential for maximising the potential of digital health technologies (42). Digital navigators can promote the use of digital technologies by offering technical support, app evaluation, and engagement strategies, thus adding a human touch to the digital transition (41). Factors promoting acceptance include perceived usefulness, self-efficacy, privacy concerns, ICT knowledge, and support seeking (43). Trust-in-use, cognitive biases, and technological expertise, alongside technological characteristics like explainability and adaptability, heavily influence medical professionals' interactions with digital and automated systems (44). Without adequate training, there is a risk of widening existing healthcare disparities, as the digital divide continues to disadvantage already marginalised groups (45).

## 5 Limitations

This study has several limitations. Participant variability in terms of professional background, experience, and exposure to digital technologies may have influenced the insights gathered. Among early adopters, exposure was not consistent—some had only recently started using a digital platform at the time of the interviews, while others had been involved in the programme for several months. Also, they were exposed to only one commercially available product (Viso—OMRON Healthcare). Additionally, the study did not account for factors such as individual attitudes toward digital health technologies, levels of seniority, training received, or familiarity with comparable systems. As an exploratory study, these variables were beyond the scope of analysis but should be systematically examined in a larger, future study. Although the study focused on a single commercially available platform, the findings primarily reflect platform-agnostic human factors and workflow considerations; nevertheless, further multi-site studies involving multiple digital solutions are needed to assess transferability and scalability across diverse NHS primary care contexts. Finally, this study focused exclusively on healthcare professional perspectives and did not include patient input, which limits insight into patient acceptance, digital literacy barriers, and equity-related factors that are critical to the successful implementation of remote monitoring technologies.

## 6 Conclusion

The integration of digital health technologies into hypertension management pathways offers significant opportunities but also presents important challenges. Digital platforms have strong potential to enhance patient engagement, improve monitoring and prioritisation, and support more accurate clinical decision-making. However, realising these benefits requires overcoming systemic barriers. Without targeted strategies, digital innovations risk widening existing health inequalities rather than reducing them. Adopting a human-centred, system-wide approach is essential to maximise the impact of digital technologies in LTC disease management and to drive a shift towards more decentralised models of care.

## Data Availability

The raw data supporting the conclusions of this article will be made available by the authors, without undue reservation.
